# Evaluation of different point-of-care tests to characterize the vaginal discharge of sows after parturition and parameters’ correlation with subsequent reproductive performance

**DOI:** 10.1186/s40813-021-00217-y

**Published:** 2021-05-20

**Authors:** A. Grahofer, T. Mäder, H. Nathues

**Affiliations:** Clinic for Swine, Department for Clinical Veterinary Medicine, Vetsuisse Faculty, University of Bern, Bremgartenstrasse 109a, CH-3012 Bern, Switzerland

**Keywords:** parturition, uterine involution, postpartal disorders, birth help, vaginal pH

## Abstract

The lochia is the physiological uterine discharge post-partum, whereas abnormal fluids are often indicators of puerperal disorders in sows, which negatively influence the further reproductive performance. The aim of the study was to characterize the vaginal discharge in sows employing simple and feasible tests and to correlate the evaluated parameters with the subsequent reproductive performance of these sows. The birth process of 48 clinically healthy free farrowing sows was monitored and several parameters characterizing the vaginal discharge such as total amount, colour, amount of cells (somatic cell count) and cell characteristics (cytology) were collected daily from first to fifth day after parturition. Finally, the reproductive performance of the following gestation was evaluated and compared to the characteristics of the lochia. The amount of vaginal discharge was significantly increased on the second (p < 0.01), third (p = 0.019) and fourth (p = 0.011) day post-partum compared to day one. Furthermore, a decrease in the percentage of neutrophilic granulocytes from day one to three (p = 0.038), four (p = 0.038) and five (p = 0.048) post-partum was observed. The percentage of neutrophilic granulocytes in the yellowish vaginal discharge was increased compared to whitish (p = 0.02) or clear (p = 0.027) vaginal discharge. In addition, obstetrics (p = 0.003) and an increased farrowing duration (p = 0.017) significantly increased the amount of vaginal discharge. Sows with a high amount of vaginal discharge had a significant higher body temperature than sows with no (p = 0.014) or low amount (p < 0.01) of vaginal discharge. No correlation was detected between the evaluated parameters of the lochia and the subsequent reproductive performance. It is hypothesised that the amount of vaginal discharge alone is not a predictor for the performance of sows during their next gestation. However, it might serve as indicator for acute endometritis. In summary, the different parameters of the vaginal discharge determined by means of point-of-care tests might be useful to strengthen a presumptive diagnose of endometritis in sows during the first five days after parturition.

## Introduction

Over the past three decades, the reproductive performance of sows has been considerably enhanced, due to genetic selection and improvement of housing and health management [1, 2]. The number of weaned piglets per sow per year has risen from 20 to 30 and may increase even more in the future [1]. It is undeniable that a large litter size causes prolonged farrowing duration and decreases the health of the sows [2].

The farrowing procedure physiologically initiates an inflammatory process to enhance uterine clearance [3]. However, nowadays, prolonged parturition duration in hyperprolific sows increases the incidence of postpartal disorders, especially endometritis [4], and thereby negatively affects the subsequent reproductive cycle and performance of the sows [5, 6]. Hence, the health status of breeding sows in the postpartal period is critical for a good reproductive performance of the herd, has a major impact on animal welfare of sows and their piglets, as well as on the economic situation of the farm [1]. It is indisputable that a diagnostic approach is required to identify pathological disorders at an early stage of any disease.

Several studies have reported that post-partum vaginal discharge occurred frequently in healthy and diseased animals [7–13], with the highest incidence between day 2 and 4 post-partum. Physiological vaginal discharge, which is watery or slightly cloudy, can be observed immediately after parturition [14–17]. An increased volume of vaginal discharge in sows is associated with endometritis, but there is no association between the occurrence of endometritis and the colour of the vaginal discharge [14, 17]. Risk factors, such as obstetrical intervention and prolonged parturition, increase the amount of vaginal discharge during the puerperium [10, 12, 18] and lead to a higher incidence of endometritis in sows [6].

Although characteristics of the sows’ lochia after parturition, such as amount, color and pH-value, have been described a few times in the literature, only fragmented data is available. Furthermore, most of these studies assessed the lochia in breeds with low fertility and not in modern hyperprolific sows. In particular, specific information regarding these parameters in free farrowing sows with high fertility, as shown by today’s genetic lines, is not available. Therefore, the aim of this study was to characterize the lochia in sows using simple and feasible test methods during five days after parturition and to correlate these parameters with the subsequent reproductive performance of the sows.

## Materials and methods

### Animals, husing and management

This observational study was conducted in a farm with free farrowing pens, as part of a sow pool system. In total, 48 healthy Large White x Landrace sows, with a mean body condition score (BCS) of 3.4 ± 0.3 were included and split into three consecutive farrowing batches. The BCS of the sow was evaluated visually on an ordinal scale ranging from 1 to 5 always by the same investigator [19]. The sows were transported to the farrowing unit approximately 7 days before their estimated farrowing date (115 days of gestation) and were housed in farrowing pens, measuring 2.2 × 2.6 m (5.72 m2), part of which was a 2.75 m^2^ slatted floor. The room temperature in the farrowing unit was approximately 22 °C. The sows were fed twice a day with a commercial feed (digestible energy 13.8 MJ/kg, crude protein 22.0 %, crude ash 7.8 %, crude fat 6.0 %, crude fibres 5.2 %) via a liquid feeding system. The sows had free access to water, which was offered through bowl drinkers. In addition, straw was provided daily to encourage rooting behaviour (approximately 1 kg/sow/day). The study protocol was approved by the cantonal veterinary office of Solothurn (Licence Nr. SO 18/01 29,818).

### Data collection

**Fig. 1:**
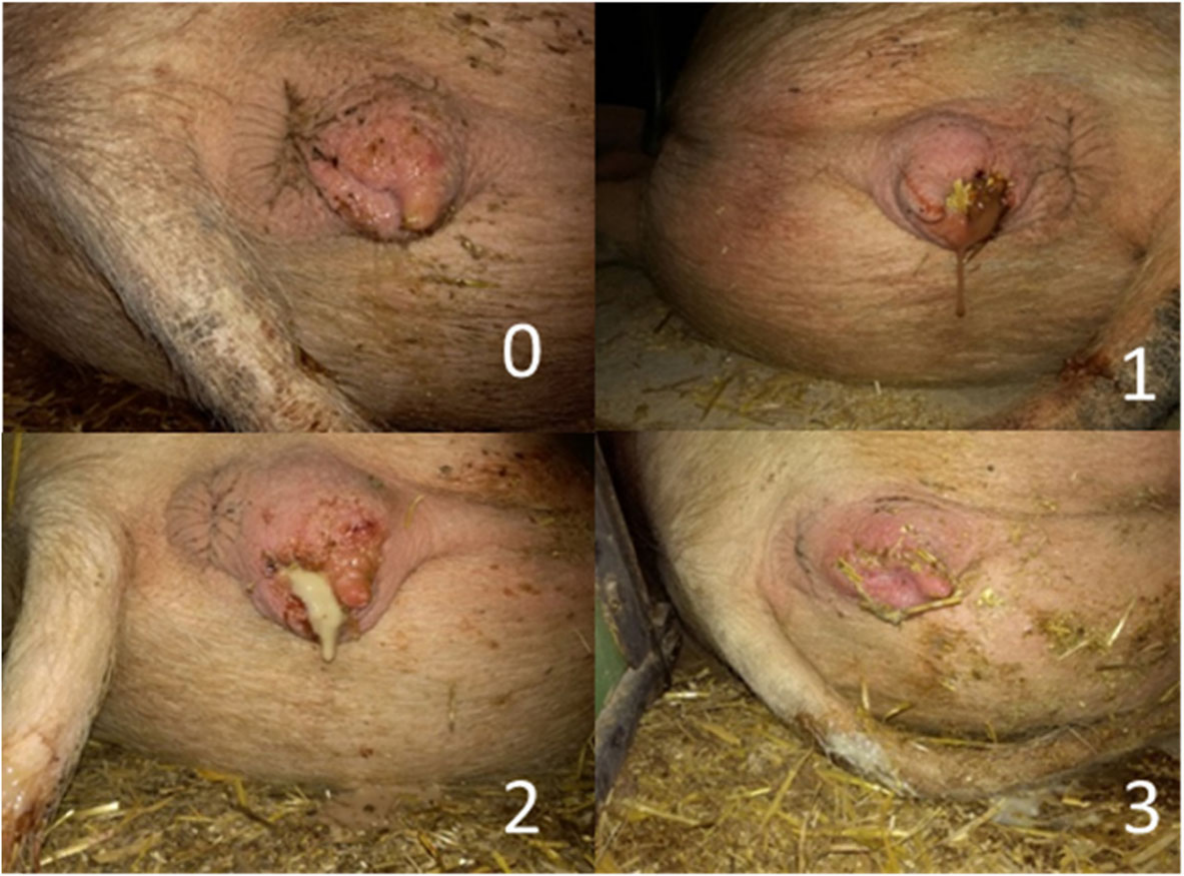
Colour score of puerperal vaginal discharge. 0= clear, 1= reddish, 2=yellowish and 3= whitish

Before parturition, the sows in each of the three farrowing batches were assigned to one of four parity groups: Group A: parity 1, group B: parity 2 and 3, group C: parity 4 and 5, group D: parity ≥ 6 in order to account for parity effect(s) of the evaluated parameters. Furthermore, sows that received a birth induction with PGF2α (10 mg *Dinoprostum* intramuscular) were recorded. PGF2α was intramuscularly injected on day 116 of gestation in case a sow had not farrowed until then. The data collection regarding the parturition was initiated with expulsion of the first piglet. The whole farrowing process was monitored at least every twenty minutes and data was collected. At each observation, the pen was checked for newborn piglets and placenta parts in order to retrospectively determine the duration of farrowing (first piglet to last placenta). In addition, obstetrical intervention was recorded, which was conducted if the piglet-to-piglet interval was longer than 60 min. Obstetrical intervention included manual uterine exploration and manual extraction of piglets followed by an intramuscularly injection of 20 IU of oxytocin. With the expulsion of the last placenta, the data collection of the different parameters post-partum started. Therefore, every morning before feeding started, the vaginal discharge was examined and the quantity, colour and pH-value was evaluated until day five post-partum. Furthermore, a cytological smear was conducted, and the number of somatic cells were evaluated. In addition, the body temperature of the sow was measured and the feed intake was assessed. Feed intake was evaluated by visual examination one hour after feeding on a dichotomous scale (normal feed intake: complete feed intake; reduced feed intake: partly or no feed intake). The quantity of the vaginal discharge was categorized into score 0 (no signs of vaginal discharge), score 1 (slight vaginal discharge) and score 2 (severe vaginal discharge, tail and perineal region contaminated with vaginal discharge). The colour of the vaginal discharge was classified into clear, whitish, yellowish and reddish (Fig. 1). For the evaluation of the pH-value, two different test methods were applied. First, a pH-indicator paper (Merck Millipore, Darmstadt-Germany) was used to evaluate the pH-value of the vaginal discharge. The pH-indicator paper was inserted into the vagina of the sow and the result was analysed and recorded immediately after removal. The second test for assessing the pH-value was based on a cotton swab (EcoCareTM comfort, Merete Medical GmbH, Berlin-Germany), which is often used in women for early detection of vaginitis, and is able to measure pH-values ranging from 4.0 to 7.5. The swab was inserted into the vagina of the sow and the result was analysed and recorded directly after removal.

The PortaSCC® Quick test (PortaCheck, Moorestown- United States), which has been established to determine the cell content of milk in dairy cows, was used to determine the somatic cell count of the vaginal discharge. The test was used according to manufacturer’s instruction and results were recorded based on the corresponding scoring scheme. This allows measuring somatic cell estimates in a unit ‘x 1000 cells/ml’ that can also be expressed on an ordinary scale: 1 (< 100); 2 (250); 3 (500); 4 (750); 5 (1500); 6 (> 3000).

In addition, two different test systems for the cytological examination were used in this field study. A smear of vaginal discharge was rolled across a clean microscope slide and after air drying stained with Giemsa staining. Furthermore, rapid staining technique, Testsimplets® (Waldeck, Münster, Germany) was used. The vaginal discharge on a swab was rolled across the slides. Since the vaginal discharge was viscous, the slides had to be rinsed with distilled water before interpretation. The water led to dissolution of the coating and staining of the cells. All slides were evaluated with a light microscope in a 400-fold magnification and cells were counted in ten randomly chosen visual fields. Cells were categorized into epithelial cells and leukocytes. Epithelial cells were further differentiated into basal cells, parabasal cells, intermediate cells and superficial cells. Leukocytes were categorized into macrophages, lymphocytes, neutrophilic granulocytes, basophilic granulocytes and eosinophilic granulocytes. For the interpretation of the data, proportions between different cells were calculated. A distribution between epithelial and all nucleated cells, between leukocytes and all nucleated cells, and between neutrophilic granulocytes and all nucleated cells was considered.

The subsequent reproductive performance of sows was assessed from the farm’s computerized production report. Thereby, the percentage of sows returning to oestrus within six weeks after insemination, the total born piglets and live born piglets were evaluated.

### Statistical analysis

Data were collected using structured and standardized data collection forms. All data were entered into a spreadsheet program (Microsoft Office Excel 2010). Statistical processing of all data was done in NCSS 12 Data (NCSS 12 Statistical Software (2018). NCSS, LLC. Kaysville, Utah, USA, ncss.com/software/ncss). Normality of continuous data was assessed using the Shapiro-Wilk test. For non-normally distributed and categorical data, the Kruskal-Wallis One-Way ANOVA was employed in more than two compared groups and the Mann-Whitney U test in two compared groups. Additionally, the Tukey Kramer Multiple-Comparison test was used for distinguishing the groups that were different from each other. For normally distributed data with equal variance the T-test was chosen. For binary data the Pearson’s Chi Square Test or for 2 × 2 field tables the Fisher’s Exact Test was employed. The correlation between the amount of vaginal discharge and the subsequent reproductive performance was analysed. Sows were assigned to either a high amount of vaginal discharge (HIGH) group or a low (LOW) group. The threshold for the HIGH group was set to the sum of all scoring values over the 5 days at ≥ 3 because this was the mean value of all sows in this study. In all tests the level of statistical significance was considered being p < 0.05.

## Results

### Descriptive statistics

**Fig. 2:**
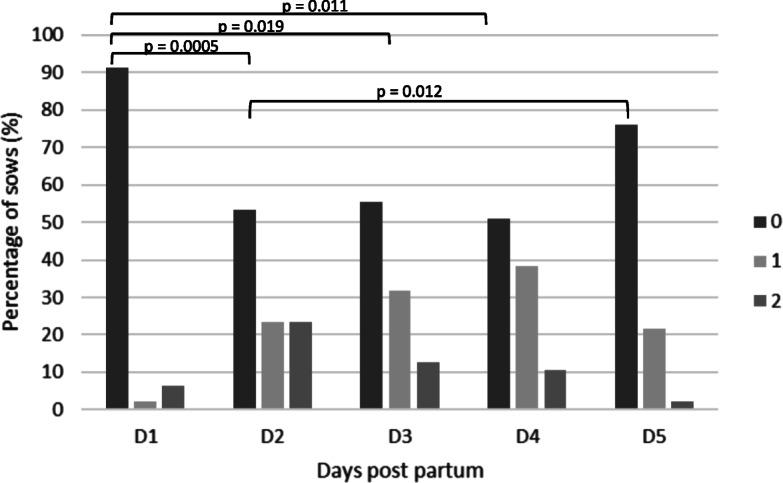
Distribution of the percentage of sows according to the quantity of vaginal discharge (0= no signs of vaginal discharge, score 1= slight vaginal discharge and score 2= severe vaginal discharge, tail and perineal region contaminated with vaginal discharge) from day 1 to day 5 after parturition

**Fig. 3:**
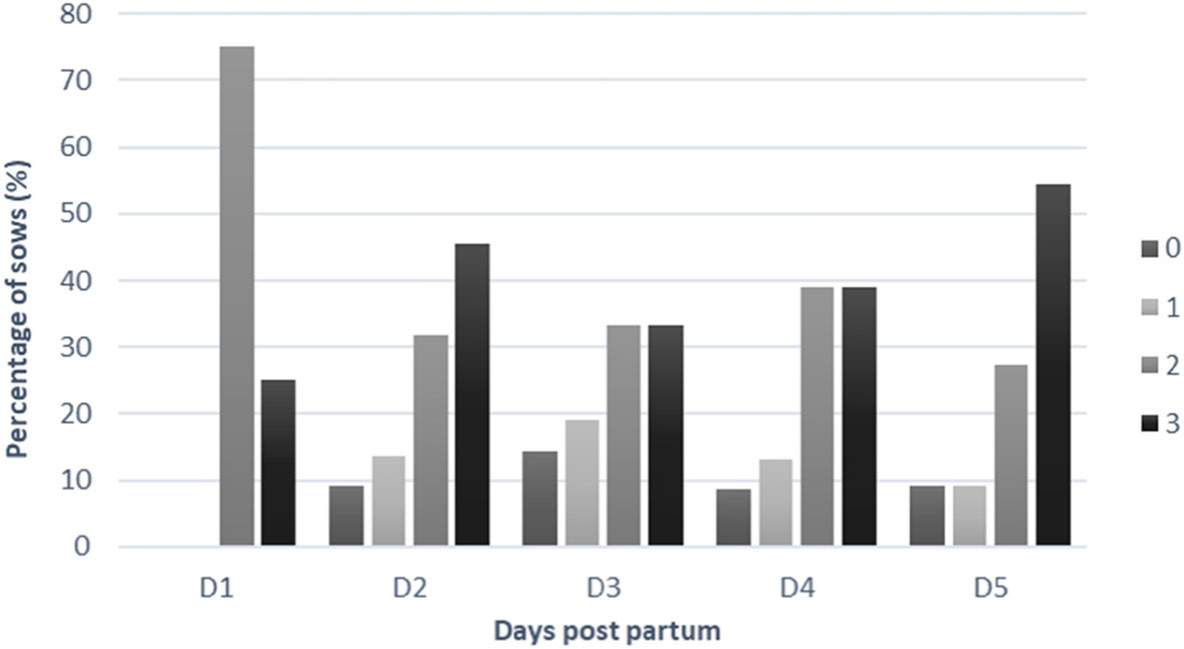
Distribution of the percentage of sows with vaginal discharge according to colour of vaginal discharge (0= clear, 1= reddish, 2=yellowish and 3= whitish) from day 1 to day 5 after parturition

In total, 48 sows were included in this study. The mean parity of the study population was 3.6 ± 2.5 (mean ± standard deviation) and ranged from 1 to 11. The mean number of total born piglets was 14.8 ± 3.0, whereof 4.1 % were stillborn. Overall, 81 samples of vaginal discharge were evaluated from 39 out of 48 sows in the early post-partum period (day 1 to 5). In the other nine sows vaginal discharge could not be observed at any time point. The prevalence of post-partum vaginal discharge was significantly lower on the first day post-partum (4/48 sows; 8.3 %) compared to the second (23/48 sows; 48.0 %; p = 0.0005), third (21/48 sows; 43.8 %; p = 0.019), and fourth (22/48 sows; 45.8 %; p = 0.011) day. Further details of the prevalence of vaginal discharge are presented in Fig. 2. The evaluation of the colour of the vaginal discharge revealed that whitish vaginal discharge was most often detected with a prevalence of 40.7 % (n = 33), followed by yellowish 35.8 % (n = 29), reddish and clear 13.6 % (n = 11), 9.9 % (n = 8), respectively. Further details of the distribution of the colour of vaginal discharge on the different sampling days are presented in Fig. 3. No significant difference between the colour and the amount of vaginal discharge was detected.

**Fig. 4:**
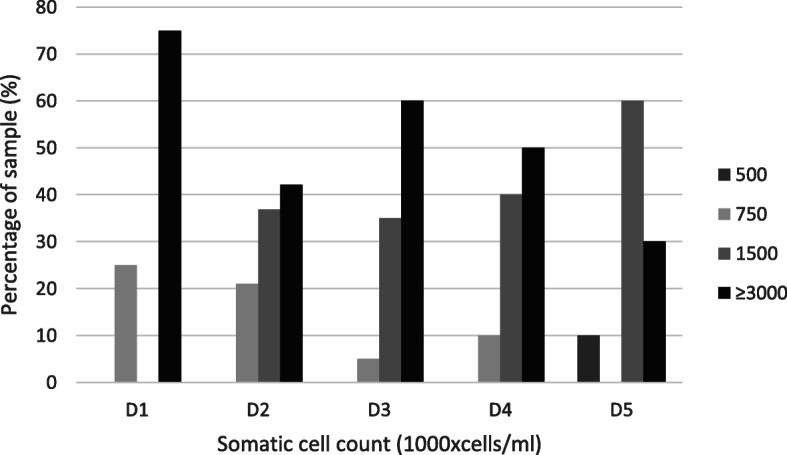
Distribution of the percentage of samples collected from sows with vaginal discharge according to the somatic cell count of the lochia tested with PortaSCC® test from day 1 to day 5 after parturition

The PortaSCC® test was conducted in all sows demonstrating vaginal discharge and detailed results are displayed in Fig. 4. The somatic cell count did not significantly differ between the days of observation. Furthermore, no correlations between the somatic cell count and the amount or the colour of vaginal discharge or the vaginal pH-value were detected.

**Fig. 5:**
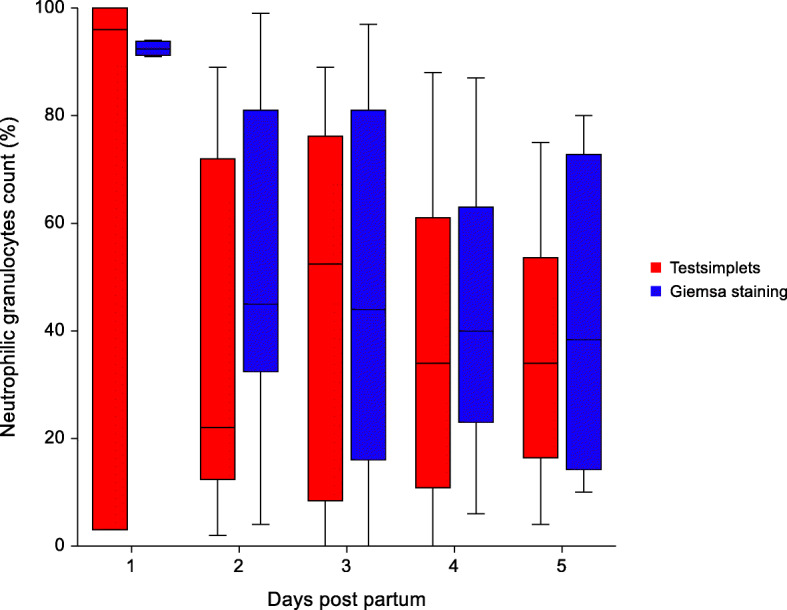
Distribution of neutrophilic granulocytes in the vaginal discharge of sows evaluated with two different methods (Testsimplets and Giemsa staing) from day 1 to day 5 after parturition. The leukocytes are expressed as a percentage of the total number of nucleated cells counted

**Fig. 6:**
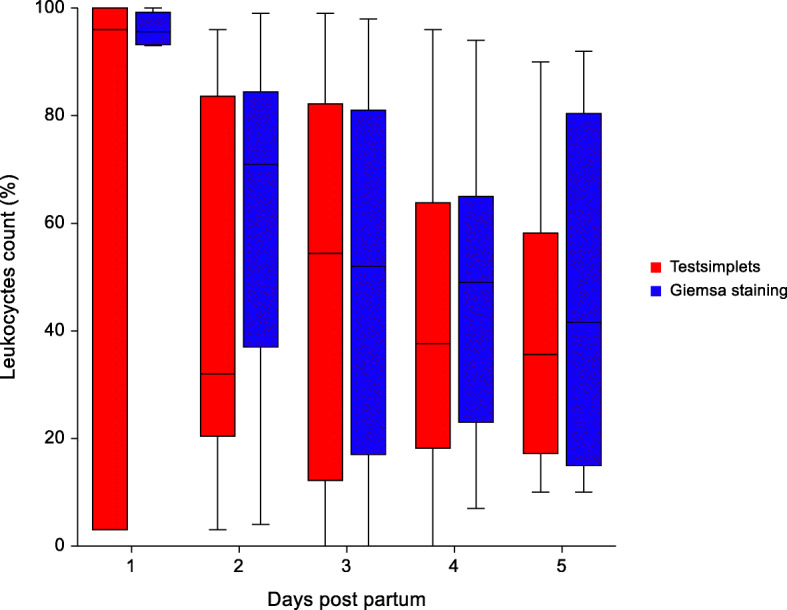
Distribution of leukocytes in the vaginal discharge of sows evaluated with two different methods (Testsimplets and Giemsa staing) from day 1 to day 5 after parturition. The leukocytes are expressed as a percentage of the total number of nucleated cells counted

**Fig. 7:**
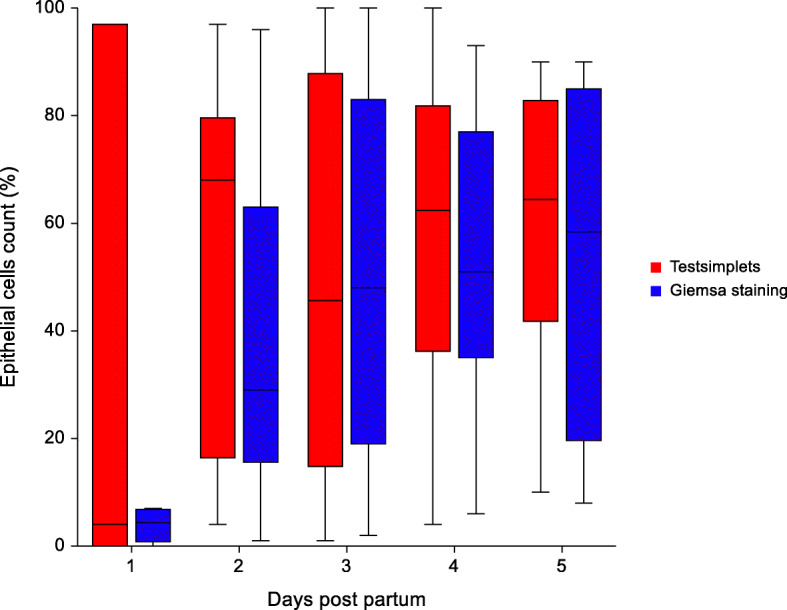
Distribution of epithelial cells in the vaginal discharge of sows evaluated with two different methods (Testsimplets and Giemsa staing) from day 1 to day 5 after parturition. The leukocytes are expressed as a percentage of the total number of nucleated cells counted

Due to a lack of sufficient amount of material to perform both smears, only four samples were collected on the first day, 17 on the second day, 15 on the third and fourth day and eight on the fifth day post-partum. An overview of the results of the cytological examination conducted with two test systems on the different sampling days are presented in Figs. 5, 6 and 7. There was no significant difference between the two test methods in evaluating the amount of leukocytes, neutrophilic granulocytes and epithelial cell. Overall, a significant difference between the colours of the vaginal discharge and the mean percentage of neutrophilic granulocytes of both tests was detected. In sows with a clear vaginal discharge less neutrophilic granulocytes (20.7 % ± 5.3) could be detected in the cytology compared to sows with a reddish (53.1 % ± 38.3; p = 0.019) and yellowish vaginal discharge (55.9 % ± 24.2; p < 0.01). In addition, sows with a yellowish vaginal discharge had significantly more neutrophilic granulocytes (55.9 % ± 24.2; p = 0.014) in the smear compared to sows with a whitish vaginal discharge (37.9 % ± 24.0).

### Analysis of vaginal discharge traits on sow traits and birth performance

**Fig. 8:**
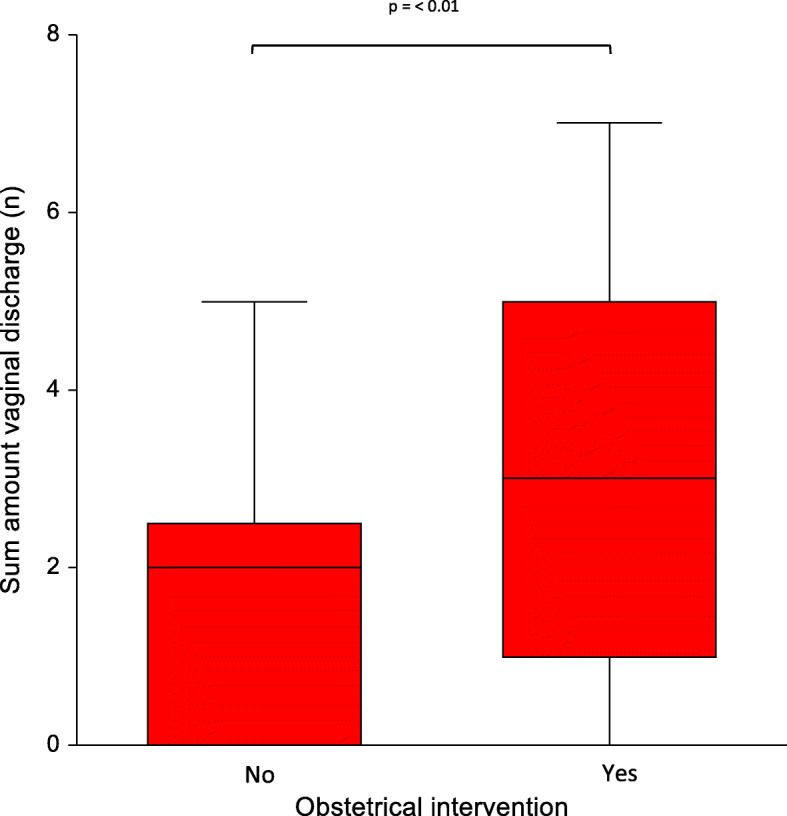
Comparison of obstetrical intervention during parturition (No= no measures conducted, Yes= obstetrics conducted) with the amount of vaginal discharge. The amount of vaginal discharge was calculated by summing the scores over the 5 days post-partum

In this study, birth induction was conducted in 16 out of 48 sows. The median farrowing duration was 211 min (Min: 55; Max: 525), without a significant difference between the parity groups. In 19 out of 48 sows (39.6 %), obstetrical intervention was conducted. Obstetrical intervention significantly (p = 0.003) increased the sum of scoring the vaginal discharge in sows within the first five days post-partum (Fig. 8).

**Fig. 9:**
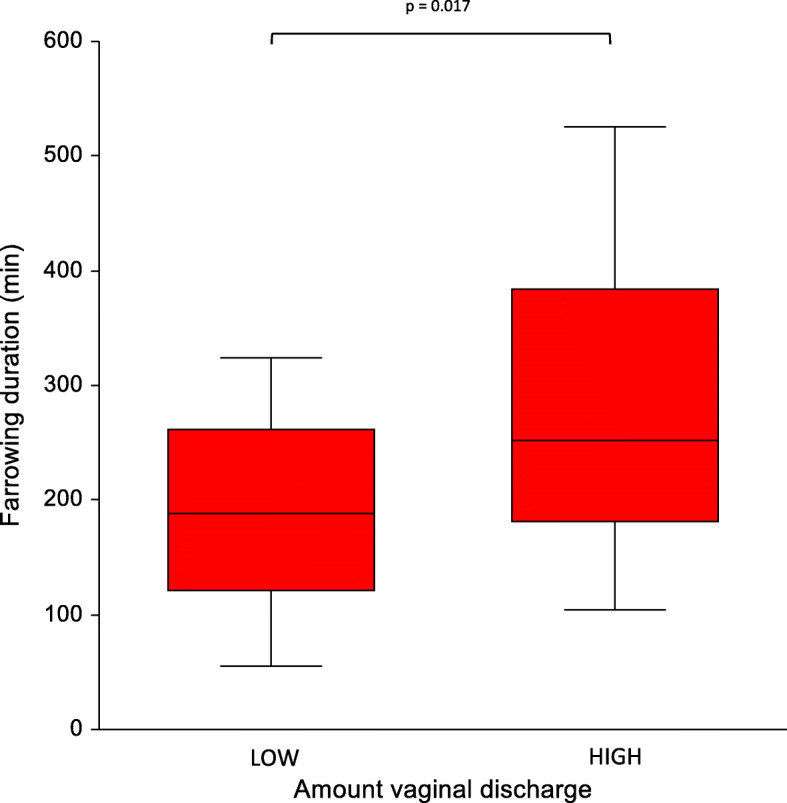
Comparison of sows that had a low amount of vaginal discharge (LOW) and sows that hat a high amount of vaginal discharge (HIGH) evaluated daily in the first five days post-partum with the farrowing duration

Retrospectively, 28 sows (58.3 %) were assigned to the low amount of vaginal discharge (LOW) group (sum of the scoring over five days ≤ 2) and 20 sows (48.6 %) were assigned to the high amount of vaginal discharge (HIGH) group (sum of the scoring over five days ≥ 3). Sows in the HIGH group showed a significantly longer farrowing duration (Median: 252; Min: 104; Max: 525) when compared to sows in the LOW group (Median: 188; Min: 55; Max: 324; p = 0.017; Fig. 9).

Further details of the study population considering parity groups, birth duration and vaginal discharge are presented in Table 1. No significant difference was detected between the LOW and HIGH group of vaginal discharge and the parity groups. Moreover, no significant differences were observed between parity, BCS, birth induction and the LOW and HIGH group of vaginal discharge.

**Table 1:** Farrowing duration clustered by parity in relation to the amount (LOW = sum of the scoring over five days ≤ 2; HIGH = sum of the scoring over five days ≥ 3) over 5 days post-partum

				Vaginal discharge					
	All			LOW			HIGH		
	Median (minutes)	Min-Max (minutes)	N	Median (minutes)	Min-Max (minutes)	N	Median (minutes)	Min-Max (minutes)	N
All sows (parity 1–11)	211	55–525	48	188.5	55–324	28	252.5	104–525	20
Parity Group A	161	104–512	13	171.5	105–277	8	161	104–512	5
Parity Group B	190	55–525	15	185	55–302	10	365	178–525	5
Parity Group C	224	96–324	8	211	96–324	6	237.5	235–240	2
Parity Group D	255	110–427	12	205	110–274	4	275	151–427	8

### Analysis of vaginal discharge traits on body temperature, feed intake post-partum and pH-value of the vagina

**Fig. 10:**
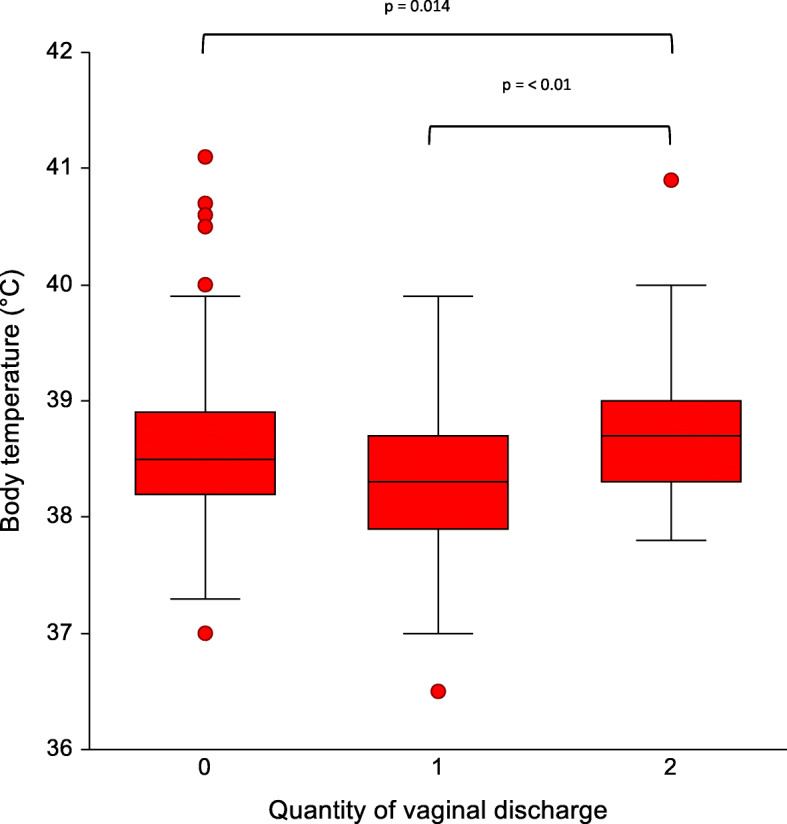
Comparison of the quantity of vaginal discharge evaluated daily in the first five days post-partum with the body temperature of sows. The quantity of the vaginal discharge was categorized into score 0 (no signs of vaginal discharge), score 1 (slight vaginal discharge) and score 2 (severe vaginal discharge, tail and perineal region contaminated with vaginal discharge)

An overview of the body temperature and the feed intake during the five sampling days grouped by the amount of vaginal discharge is presented in Table 2. No correlation between feed intake and amount of vaginal discharge on a particular sampling day was detected. A significant difference of the body temperature in sows between day 1 after parturition (Median: 38.8 °C; Min: 37.0 °C; Max: 40.7 °C) and day 3 (Median: 38.3 °C; Min: 37.5 °C; Max: 39.9 °C; p = 0.002) and day 4 (Median: 38.3 °C; Min: 37.0 °C; Max: 39.7 °C; p = 0.0018) was seen. In addition, sows with a severe amount of vaginal discharge had a significant higher body temperature (Median: 38.7 °C; Min: 37.8 °C; Max: 40.9 °C) than sows with no (Median: 38.5 °C; Min: 37.0 °C; Max: 41.1 °C; p = 0.014) or slight amount of vaginal discharge (Median: 38.3 °C; Min: 36.5 °C; Max: 39.9 °C; p < 0.01), when considering all days of observation. Details are presented in Fig. 10. Finally, no significant difference in the body temperature of sows, which received obstetrics during parturition, compared to sows without birth assistance were detected over the sampling period.

**Table 2:** Amount of vaginal discharge, body temperature and feed intake in relation to the days post-partum

Days post-partum	Vaginal Discharge	N	Temperature		Feed intake
			Median (°C)	Min-Max (°C)	% (n/N)
1	Over all	46	38.8	37-40.7	89.1 (41/46)
	0	42	38.8	37-40.7	90.5 (38/42)
	1	1	37.7		100.0 (1/1)
	2	3	38.9	38.5-38.9	66.6 (2/3)
2	Over all	47	38.5	36.5-41.1	93.6 (44/47)
	0	25	38.5	37-41.1	92.0 (23/25)
	1	11	38.5	36.5-39.9	90.9 (10/11)
	2	11	38.8	37.8-40.9	100.0 (11/11)
3	Over all	47	38.3	37.5-39.9	97.9 (46/47)
	0	26	38.4	37.5-39.9	96.1 (25/26)
	1	15	38.2	37.5-39.7	100.0 (15/15)
	2	6	38.4	38.1-38.8	100.0 (6/6)
4	Over all	47	38.3	37-39.7	95.7 (45/47)
	0	24	38.3	37.6-39.7	100.0 (24/25)
	1	18	38.2	37-39.3	94.4 (17/18)
	2	5	39	37.8-39.3	80.0 (4/5)
5	Over all	45	38.5	37.3-40.5	97.8 (45/46)
	0	35	38.5	37.3-40.5	97.1 (34/35)
	1	9	38.1	37.4-39.7	100.0 (10/10)
	2	1	40		100.0 (1/1)

**Fig. 11:**
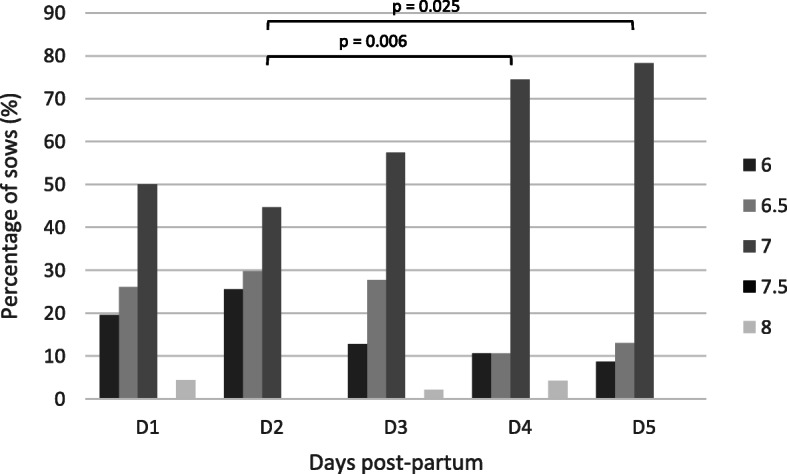
Distribution of the percentage of sows according to pH-value of the vagina tested with a strip system from day 1 to day 5 after parturition

**Fig. 12:**
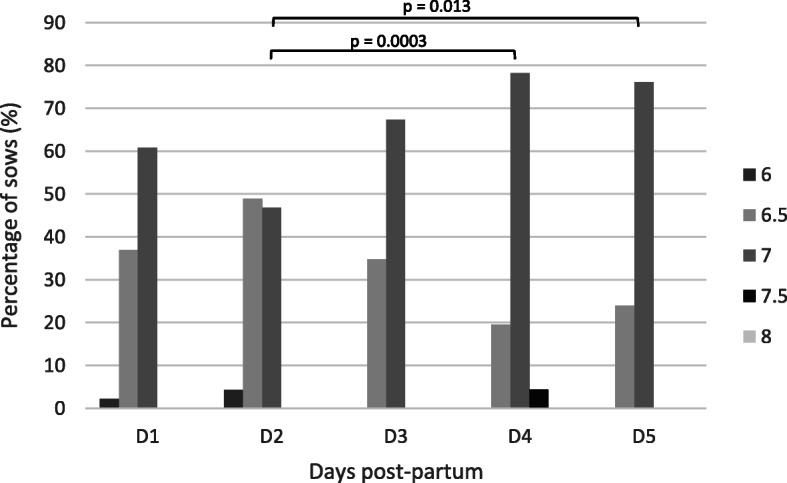
Distribution of the percentage of sows according to pH-value of the vagina tested with a swab system from day 1 to day 5 after parturition

The vaginal pH-value of 46 sows on first- and fifth-day post-partum and of 47 sows on the other sampling days were measured with two different test systems (strip and swab test). The data of each test system over the sampling period are presented in Figs. 11 and 12. In 74.9 % of the samples the same pH-value was obtained in the same individuum using different tests. Hence, no significant difference between the two test systems could be determined. A significant difference in the pH-values between the second (strip: 6.6 ± 0.4; swab: 6.7 ± 0.3) and fourth day (strip: 6.9 ± 0.4; swab: 6.9 ± 0.2; strip: p = 0.006 and swab: p = 0.0003) and the second and fifth day post partum (strip: 6.8 ± 0.3; swab: 6.8 ± 0.2; strip: p = 0.025 and swab: p = 0.013) was detected.

A significant difference among the pH-value of the vagina tested with the strip and the swab was detected between sows with no signs of vaginal discharge (p = 0.0118) and slight vaginal discharge (p = 0.0109). Sows with no vaginal discharge had a lower vaginal pH-value (6.7 ± 0.4; 6.8 ± 0.2) compared to sows with a slight vaginal discharge (6.9 ± 0.3; 6.9 ± 0.2) In addition, a significant difference of the pH-value tested with the strip between sows with a reddish vaginal discharge compared to sows with a yellowish (p = 0.0212) and whitish vaginal discharge (p = 0.0182) was detected. Sows with a reddish vaginal discharge had a lower vaginal pH-value (6.6 ± 0.6) compared to sows with yellowish (6.9 ± 0.4) and whitish (6.9 ± 0.3) vaginal discharge. This trend could also be observed in the pH-values obtained with the vaginal swab, but no significant difference was confirmed.

### Analysis of vaginal discharge traits on subsequent reproductive performance

In this study, 6 out of 43 sows (13.9 %) returned to oestrus within six weeks after the subsequent mating. No significant difference between the return to oestrus rate could be determined among the LOW (4 out of 25 sows; 16.0 %) and HIGH (2 out of 18 sows; 11.1 %) group. The total born piglets and live-born piglets at the subsequent farrowing in sows with LOW vaginal discharge (15.0 ± 3.8; 14.3 ± 3.6) was increased compared to the group with HIGH amount of vaginal discharge (14.3 ± 3.1; 13.7 ± 3.3), but no significance could be confirmed. Furthermore, no other significant difference between the other parameters of the vaginal discharge and the subsequent reproductive performance was detected.

## Discussion

This is the first study that evaluates different point-of-care tests in parallel to characterize the lochia of sows after parturition in a free farrowing system. Furthermore, the characteristics of the vaginal discharge were associated with the subsequent reproductive performance of the same sows. The study design included a stratified random allocation blocked by parity to control the parity effect. Only the principal investigator conducted the sampling of the vaginal discharge and the post-partum parameters to reduce the observer bias. Although 48 sows were included in this observational study, only 39 sows showed signs of vaginal discharge. It might be that some sows with vaginal discharge were overlooked, because the evaluation was made once a day only. However, to minimize false negative results in sows, the observation was conducted early in the morning during the resting period of the sows to avoid urination before sampling. Since the sample size, especially per parity, was relatively small, only descriptive analyses for the parity were conducted. However, further analyses with all animals were conducted to minimize the detection bias. Another limitation of this study is that only sows from one farm in Switzerland were examined. Nonetheless, the results of this study are novel and of major interest for managing and optimizing the post farrowing phase in free farrowing sows. In addition, the sampling time points, and the evaluated test systems used in this study can certainly be implemented in sow herds with different housing systems and not only those with free farrowing.

In the present study, the prevalence of vaginal discharge throughout the different sampling days was lower compared to recent studies, although slight amount of vaginal discharge was included in the present data set too. Moreover, not only presence, but also colour of the vaginal discharge was evaluated. In this study, 8.3 % of the sows on day 1, 48.0 % on day 2 and 43.8 % on day 3 postpartum showed vaginal discharge, which is slightly differing from literature that reports a prevalence of 70.3 and 55.6 % on day 1, 53.1 and 69.1 % day 2 and 50.6 % on day 3, respectively [11, 12]. The authors hypothesize that the reason for these differences in the prevalence of vaginal discharge is due to the free farrowing housing system, although the different assessments of the prevalence of vaginal discharge might limit the comparison of the results. However, in free farrowing system, sows can better express their natural behaviour, which presumably prevents from dysfunction of the endocrine regulation and decreases the stress level during parturition compared to systems with crated sows. Furthermore, sows in a free farrowing system have a higher average oxytocin concentration during the post-expulsion compared to crated sows [20] and thereby uterine clearance directly after farrowing is more likely. In order to scientifically proof this hypothesis, further investigations are needed.

In contrast to two studies [8, 21], where post parturient vaginal discharge was associated with parity, no significant difference between the different parities was detected in this study. However, the sample size per parity group is limited, and the housing system might have influenced the results, especially on day 1, where the effect of free movement might have the greatest impact.

In accordance with previous reports [11, 12, 22], the amount of vaginal discharge positively correlated with the body temperature in sows. Physiologically, the body temperature increases and remains high during and after farrowing [23] due to nest-building activity, development of mammary glands and uterine contractions. In addition, the farrowing procedure initiates an inflammatory process evidenced by a rise of interleukins [3]. However, bacterial infections after parturition in the urogenital tract also lead to an increase of IL-6 and tumour necrosis factor alpha in the blood, causing a rise in body temperature [3]. Therefore, it can be concluded that also in free farrowing sows the amount of vaginal discharge is a feasible parameter to evaluate uterine health after farrowing.

Data of the vaginal pH-value of sows after parturition are lacking in current literature. This study observed that the pH-value of the vagina is altering during the first days post-partum. A significant difference of the vaginal pH-value between day 2 (pH = 6.6) and day 4 and day 5, respectively, (pH = 6.9; pH = 6.8) after farrowing was observed. A study in miniature pigs showed that the vaginal pH-value is neutral (pH = ~ 7) in sexually mature animals [24], like in our study on the last two sampling days. However, on the first days a more acidic pH-value was seen, which is in line with results from human medicine, revealing an acidic vaginal secretion directly after labour [21]. Sows with a reddish vaginal discharge had a more acidic vaginal discharge compared to sows with whitish and yellowish vaginal secretion. It is hypothesised that sows with whitish and yellowish vaginal discharge have a higher risk for uterine disorders, due to changes of the microbial flora and inflammatory reactions [25]. This is in line with results of the cytological investigation, especially demonstrated by the amounts of neutrophil granulocytes. It is known from literature that sows with a higher number of polymorphonuclear cells are more prone to develop a puerperal endometritis [26]. However, further investigations are needed to prove these findings on a larger scale. In case of confirmation, these parameters could be reliable for diagnostic purposes and, thereby, help to detect postpartal disorders and enable improvement in sow and piglet health.

Hence, no correlation between parameters of the vaginal discharge and farrowing traits with the cell count of vaginal discharge could be detected, the PortaSCC® test, established for milk samples in cows, might not be an adequate test kit for vaginal discharge.

Similarly to other studies, where sows were housed in crates [6, 10, 13] a longer farrowing duration (Median: 252 vs. Median: 188) leads to a higher amount of vaginal discharge in free farrowing sows. Interestingly, the median farrowing duration of sows with a higher amount of vaginal discharge in the early puerperium is beyond the established cut-off value of 300 min [5] in crated sows. Hence, it can be discussed, if the cut-off value of farrowing duration needs to be adjusted according to the housing conditions of sows. In order to scientifically evaluate the optimal farrowing duration in free farrowing sows, further investigations are warranted.

Like in previous studies, obstetrical intervention positively correlated with the amount of vaginal discharge over the five sampling days. However, no significant differences in the body temperature and feed-intake between treated and non-treated sows were confirmed. This is in contrast with current literature. A potential explanation for these discrepancies is that the housing system influences the uterine health especially that of free farrowing sows. The physical movement after parturition might positively influence uterine involution, which is also described in the human medicine [27, 28]. Hence, the free movement supports the physiological uterine clearance of the sow by hasting the uterine involution. Uterine contractility increases and leads to an elimination of intrauterine fluid, which reduces the risk of bacterial infection and consequently minimizes the risk of fever, reduced feed intake and other post-partum disorders. This assumption is in line with a recent study showing no influence of obstetrical intervention on the uterine involution evaluated with ultrasonography in free farrowing sows [29].

In the present study only a trend between the next litter size and the amount of vaginal discharge was detected, revealing that sows with a high amount of vaginal discharge have less total and/or stillborn piglets. This is in line with an older study, where only few significant differences between the reproductive performance and the duration of vaginal discharge was described [25]. However, it can be speculated that an early and adequate treatment of sow with high amount of vaginal discharge can support the uterine clearance and therefore increases the reproductive performance in the next litter.

## Conclusions

The present study provides novel findings and original results to characterise the post parturient vaginal discharge by means of several point of care tests in free farrowing sows. In comparison with the recent literature of crated sows, the prevalence of post parturient vaginal discharge in free farrowing sows is reduced, which consequently minimizes the risk for fever, reduced feed intake and other post-partum disorders. However, a prolonged farrowing duration and obstetrical intervention significantly increases the amount of vaginal discharge. Nevertheless, it seems that the amount of vaginal discharge alone is not a valuable predictor for the performance of sows during their next gestation but might be an indicator for an acute endometritis. This indicator in combination with the colour of the vaginal discharge and the vaginal pH-value might help to detect postpartal disorders early after farrowing and therefore enable improvement of sows’ and piglets’ health. In summary, the different parameters of the vaginal discharge determined by means of point-of-care tests might be useful to strengthen a presumptive diagnose of endometritis in sows during the first five days after parturition.

## Data Availability

All the data are presented in the main paper and accompanying figures.
